# Supplementary figures and images for: Sex-biased gene expression and gene-regulatory networks of sex-biased adverse event drug targets and drug metabolism genes
Source: BMC Pharmacol Toxicol. 2024 Jan 2;25:5. doi: 10.1186/s40360-023-00727-1 (PMC10763002; doi:10.1186/s40360-023-00727-1)

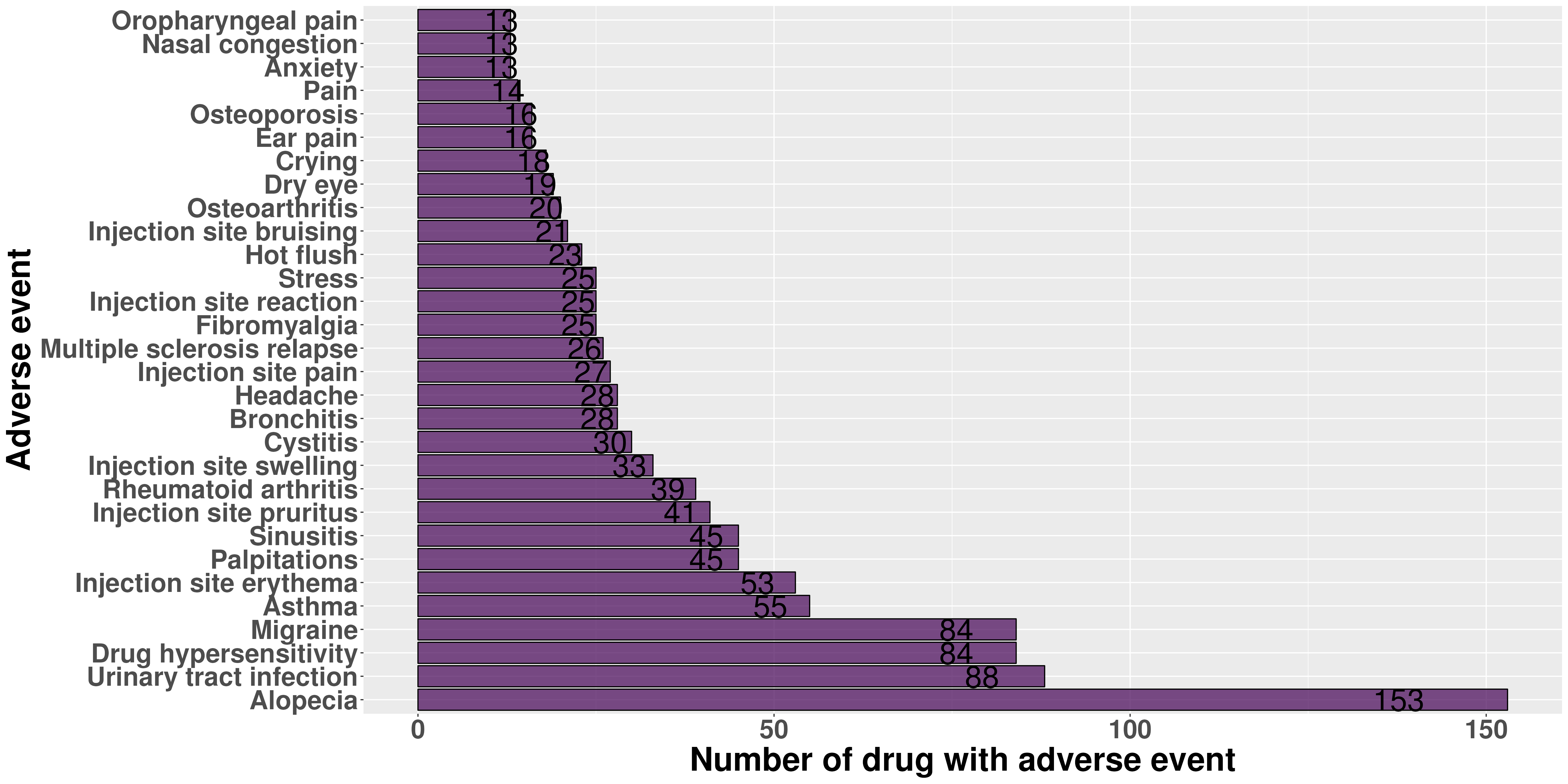

Supplement: Supplementary file 7 — Supplemental Figure 1: Barplot of the top 30 female-biased adverse events based on the number of drugs with an adverse event [file 40360_2023_727_MOESM7_ESM.png]

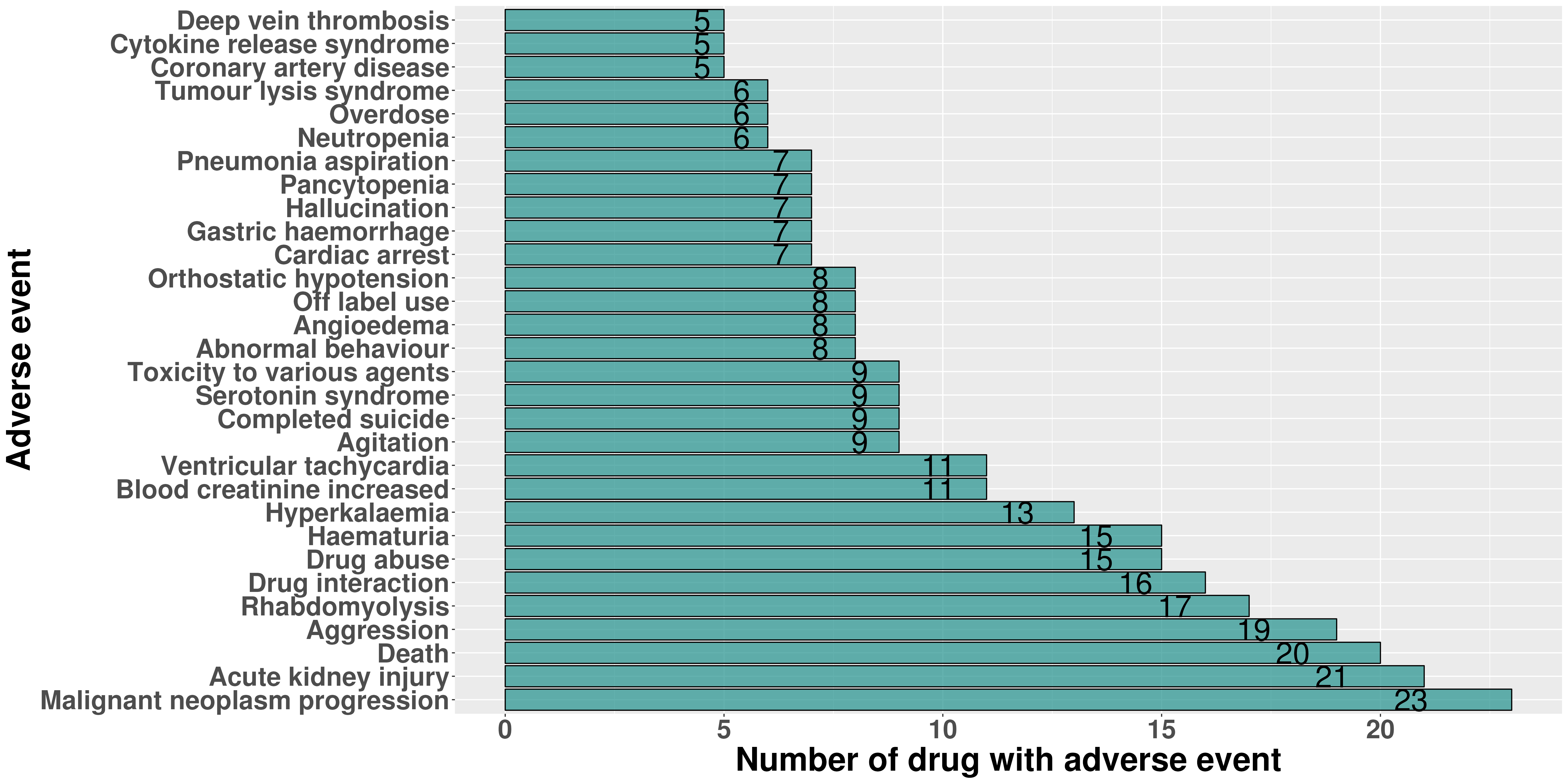

Supplement: Supplementary file 8 — Supplemental Figure 2: Barplot of the top 30 male-biased adverse events based on the number of drugs with an adverse event [file 40360_2023_727_MOESM8_ESM.png]

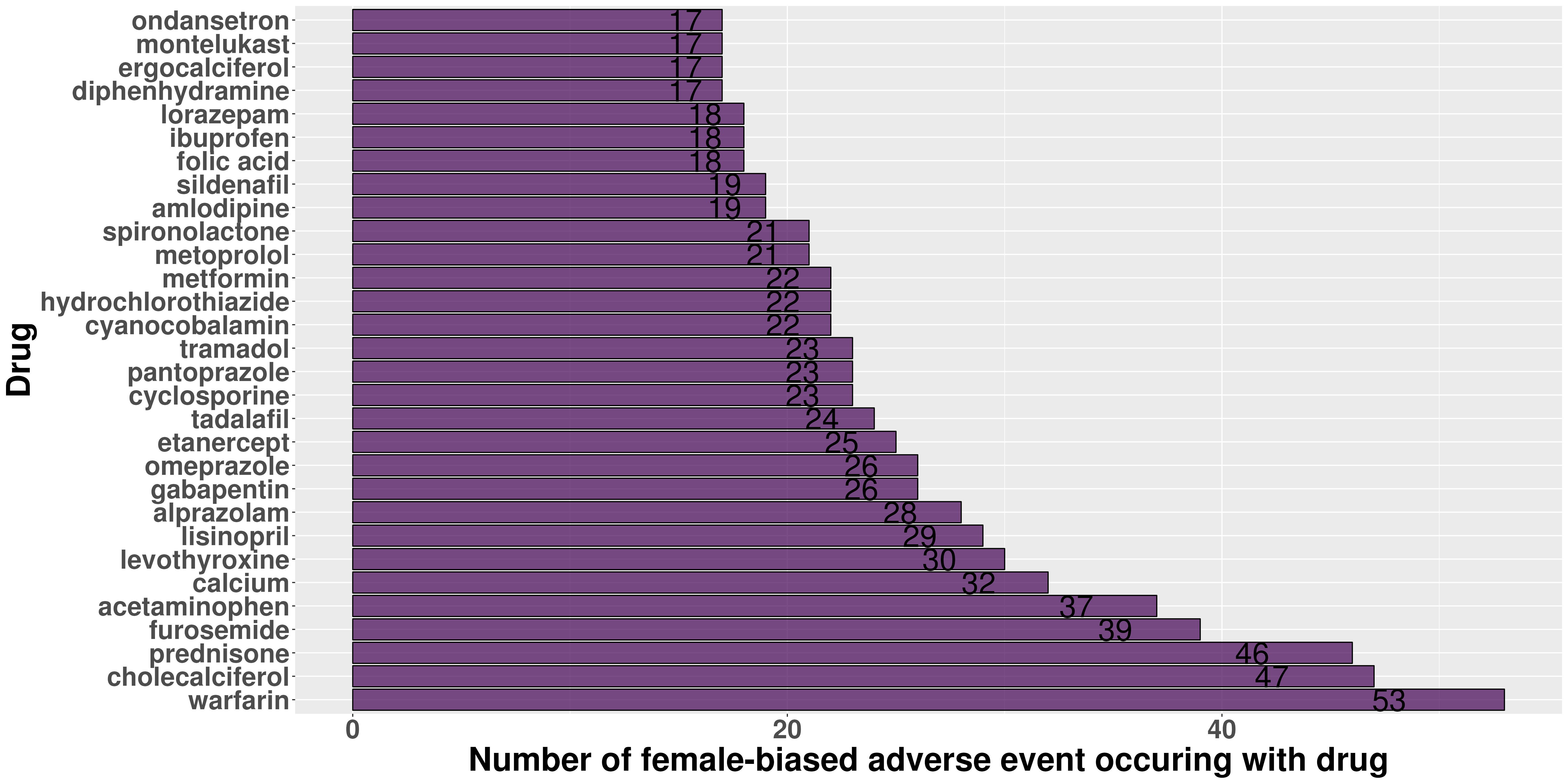

Supplement: Supplementary file 9 — Supplemental Figure 3: Barplot of the top 30 drugs based on the number of female-biased adverse events associated with the drug [file 40360_2023_727_MOESM9_ESM.png]

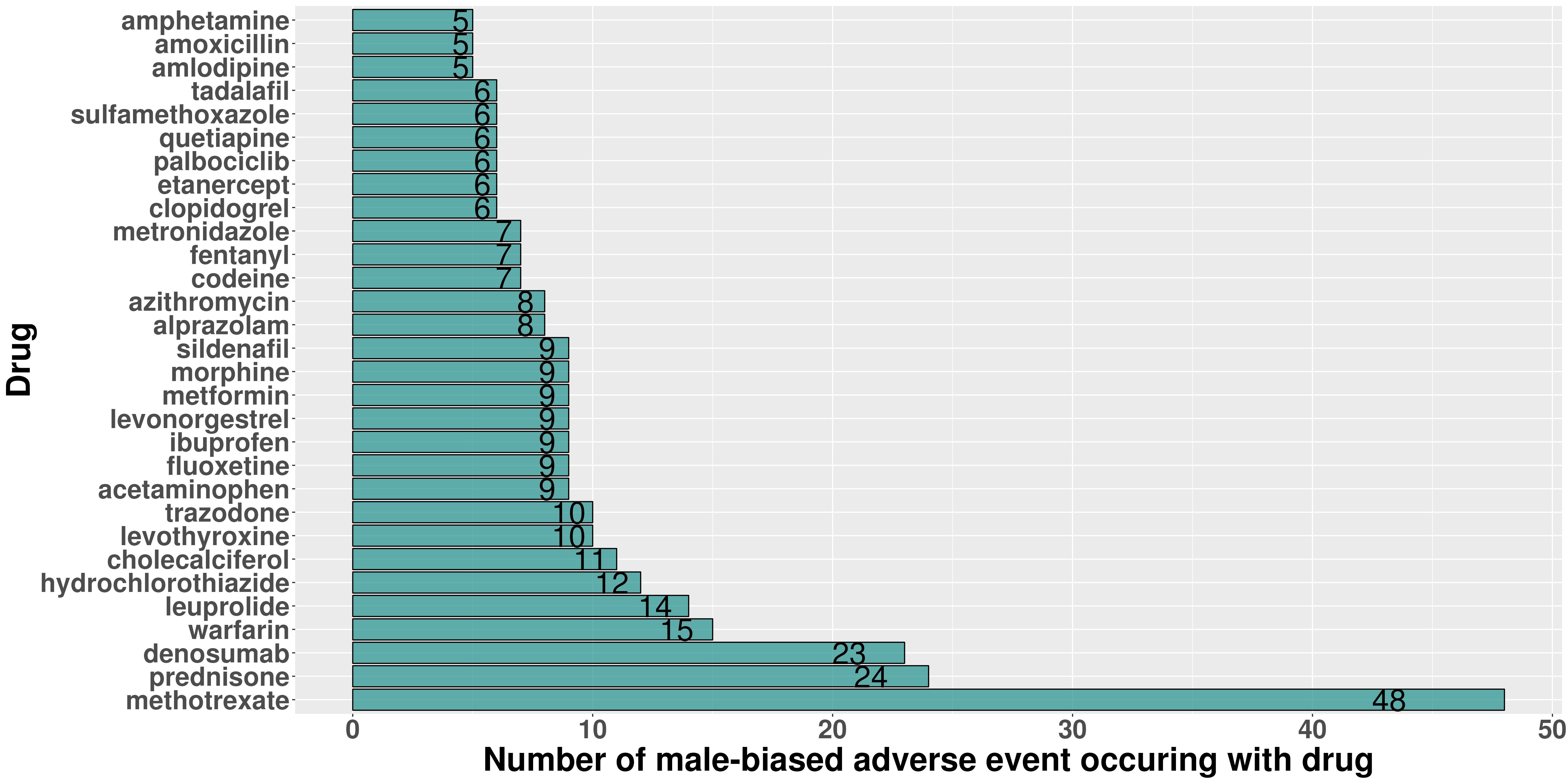

Supplement: Supplementary file 10 — Supplemental Figure 4: Barplot of the top 30 drugs based on the number of male-biased adverse events associated with the drug [file 40360_2023_727_MOESM10_ESM.png]

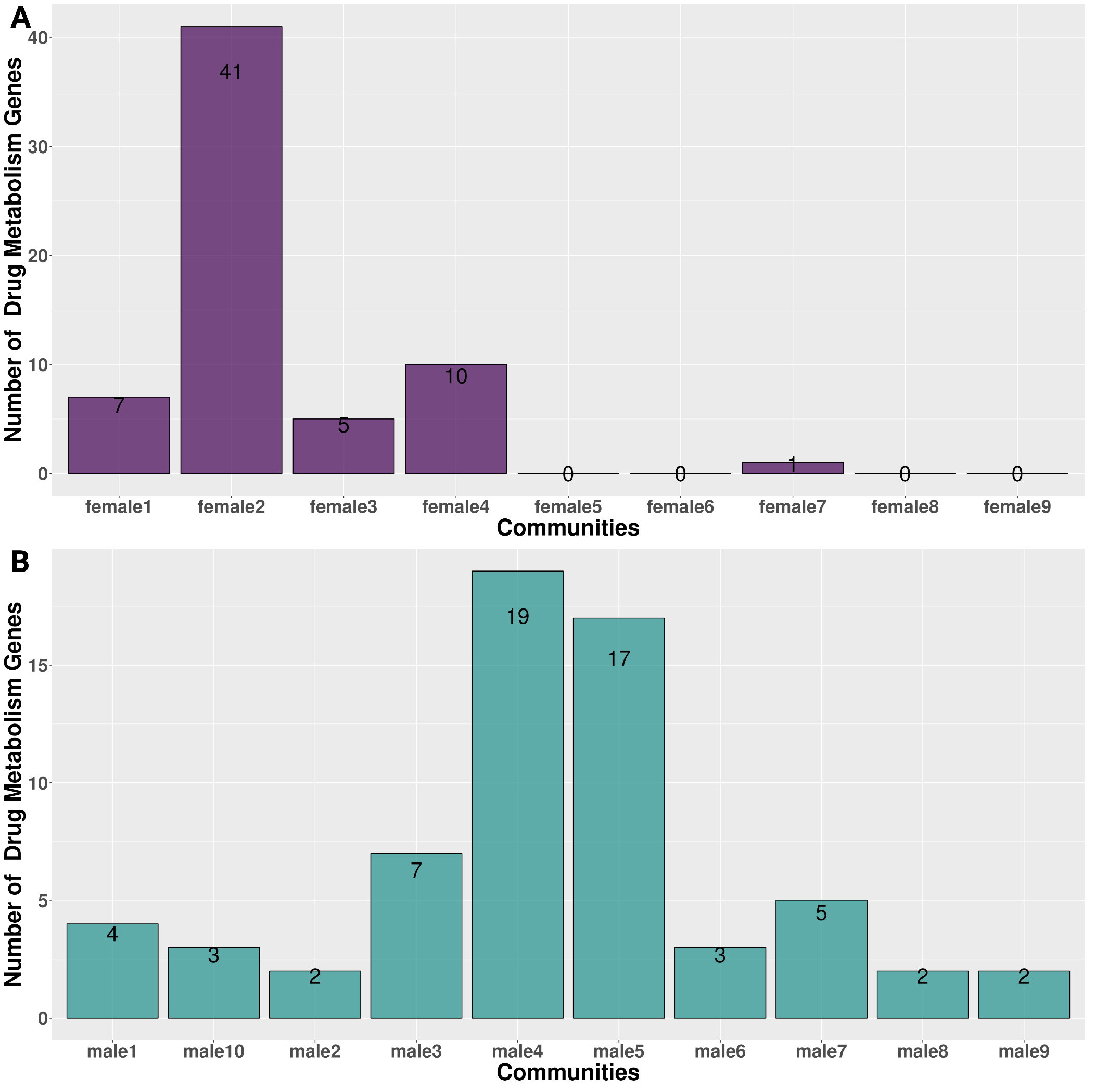

Supplement: Supplementary file 12 — Supplemental Figure 6: Bar plots of the number of drug metabolism genes in the sex-specific communities of the liver for (A) female and (B) male [file 40360_2023_727_MOESM12_ESM.png]

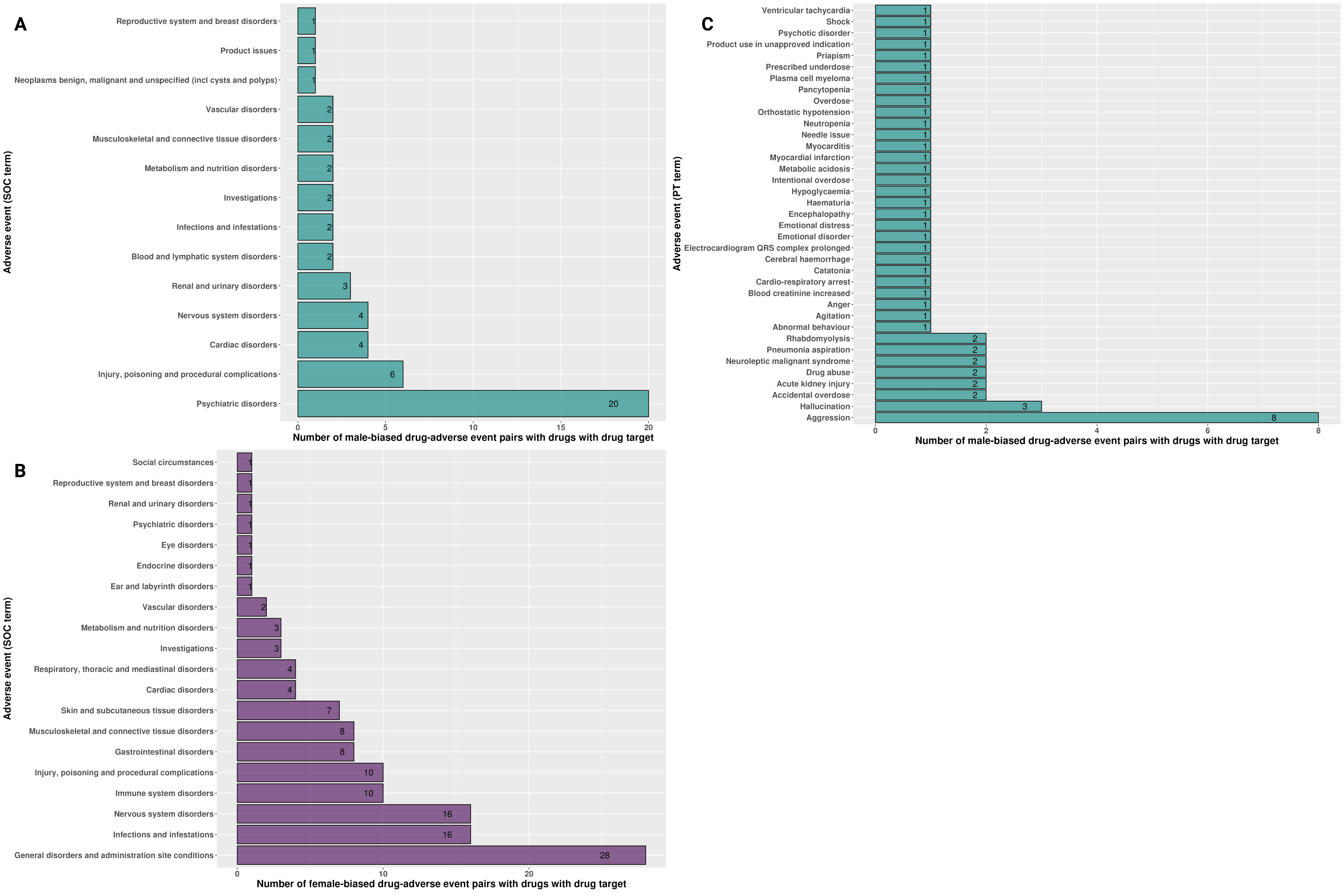

Supplement: Supplementary file 13 — Supplemental Figure 7: We performed a one-tailed Wilcoxon test to determine if the number of core genes selected for drug metabolism was higher than for the randomly selected genes for (A) Sex-biased liver core gene (p-value = 1). (B) male-specific liver core genes (p-value = 0.9910552) (C) female-specific liver core genes (p-value = 1) [file 40360_2023_727_MOESM13_ESM.png]

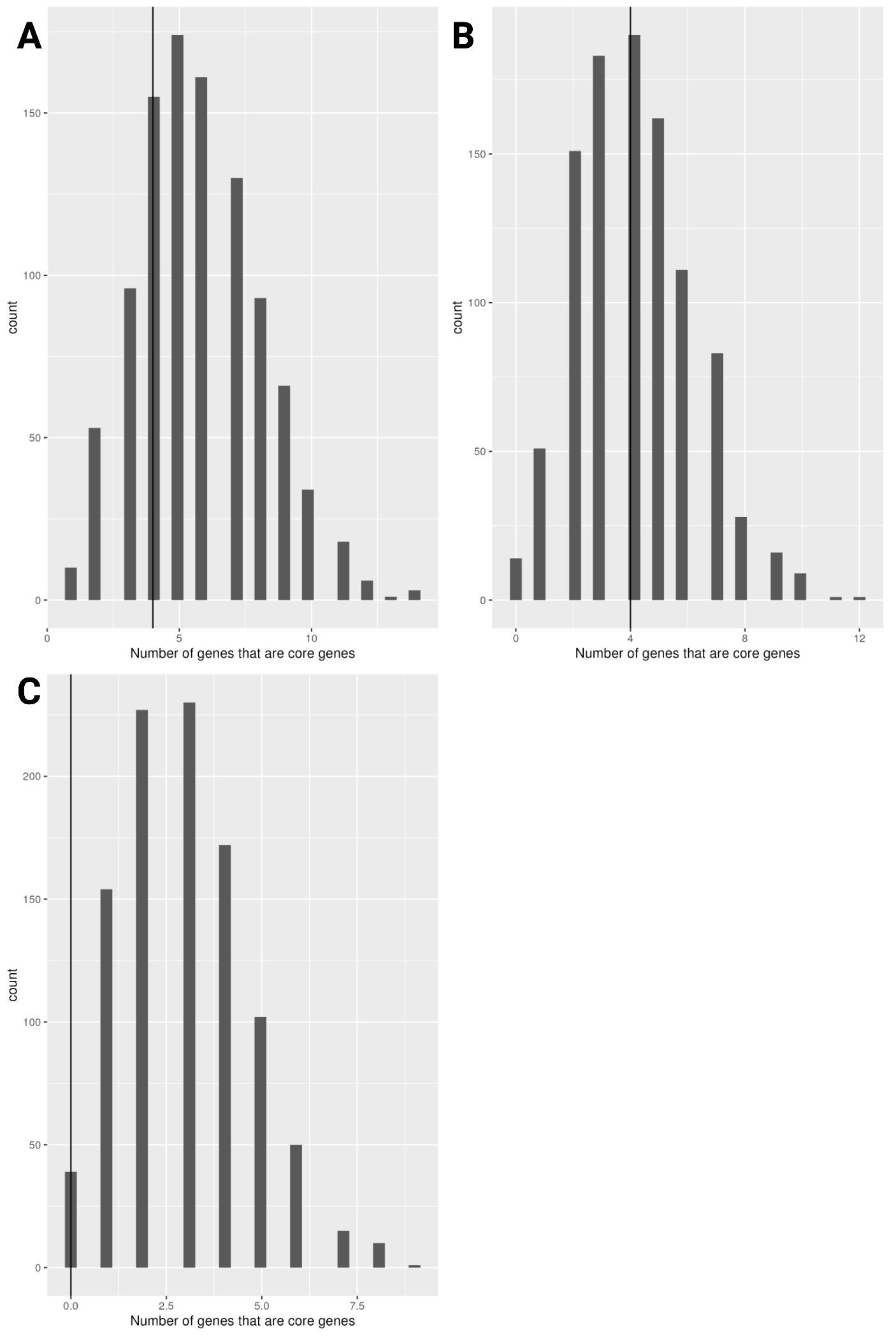

Supplement: Supplementary file 14 — Supplemental Figure 8: Permutation results for the SBAE-associated drug targets’ enrichment of sex-biased expressed genes. (A) Bar plot of the female-biased sex-biased gene expression gene sets by tissue with the x-axis being the fraction of SBAE-associated drug targets with female-biased gene expression for the tissue and the number of drug targets with sex-biased gene expression. (B) Bar plot of the male-biased sex-biased gene expression gene sets by tissue with the x-axis being the fraction of SBAE-associated drug targets with male-biased gene expression for the tissue and the number of drug targets with sex-biased expression. Permutation testing was conducted by randomly selecting either 84 drug targets genes 1,000 times with a one-tailed Wilcoxon test and with BH-multiple hypothesis test correction (α = 0.05) [file 40360_2023_727_MOESM14_ESM.png]

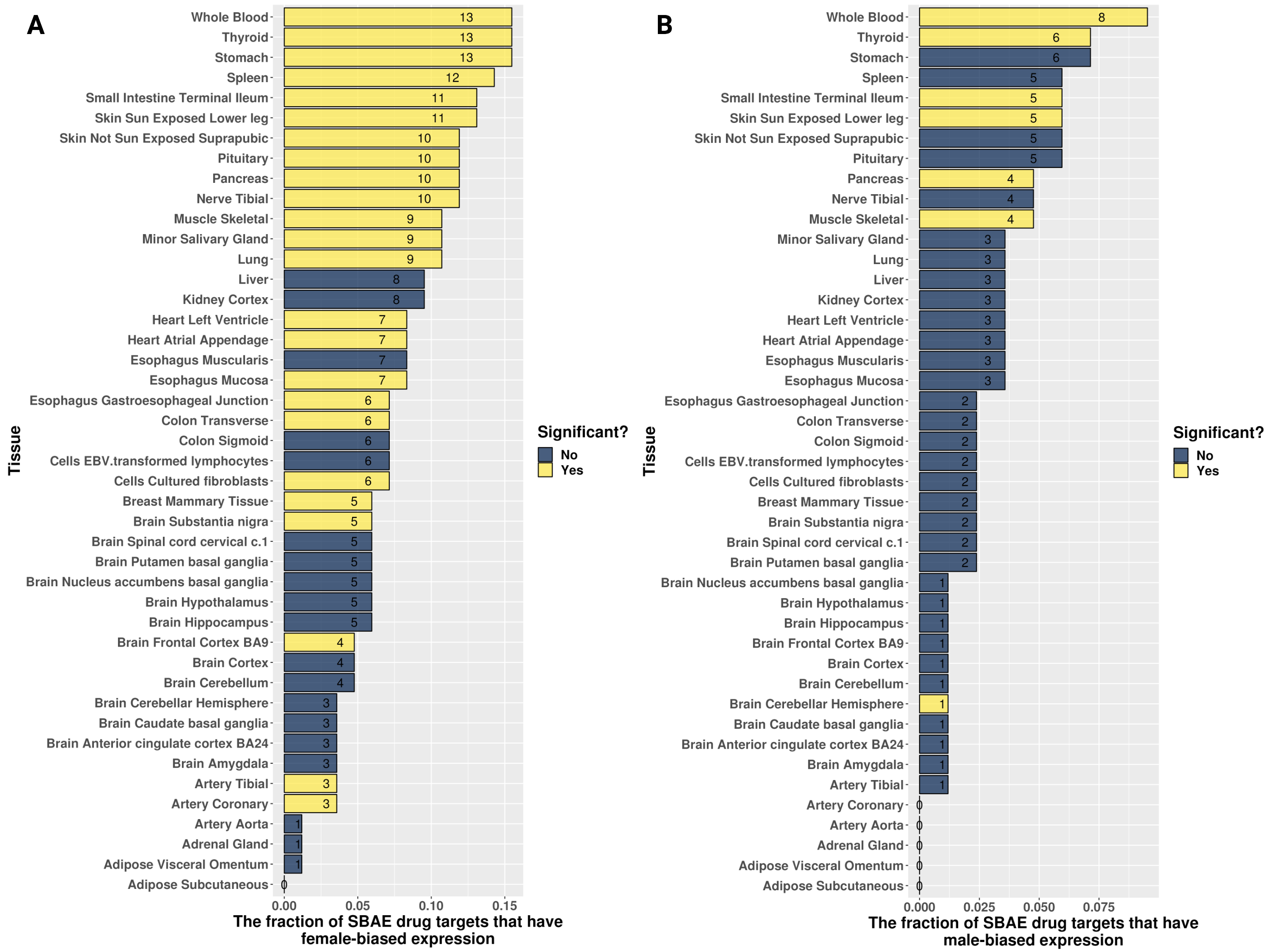

Supplement: Supplementary file 15 — Supplemental Figure 9: Drug target ADRA1A’s drug-adverse event plots. (A) A bar plot of the number of male-biased drug-adverse event pairs with a drug with ADRA1A as drug target for each System Organ Class (SOC) adverse event term. (B) A bar plot of the number of male-biased drug-adverse event pairs with a drug with ADRA1A as drug target for each Preferred Term (PT) adverse event term. (C) A bar plot of the number of female-biased drug-adverse event pairs with a drug with ADRA1A as drug target for each System Organ Class (SOC) adverse event term [file 40360_2023_727_MOESM15_ESM.png]

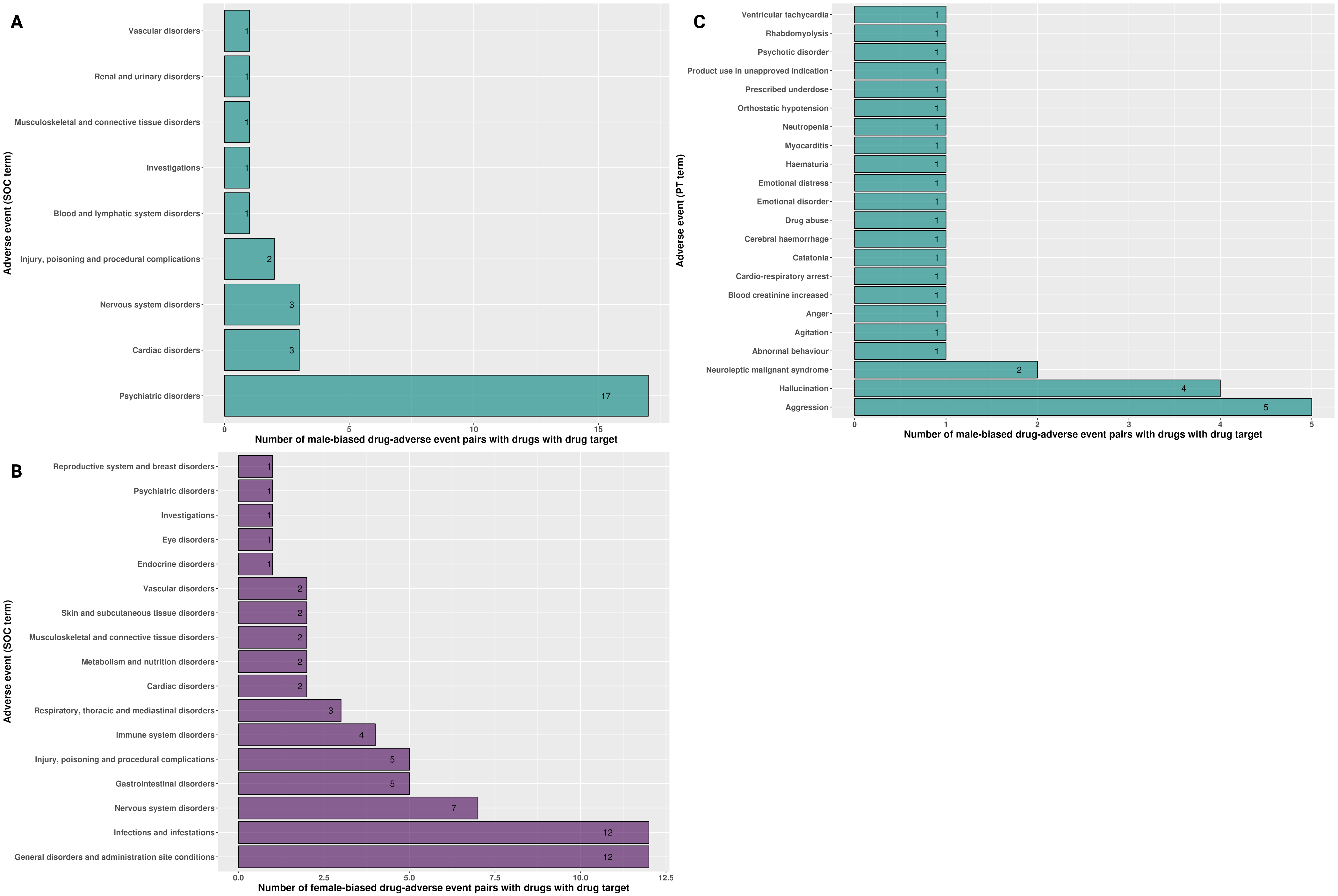

Supplement: Supplementary file 16 — Supplemental Figure 10: Drug target ADRA1C’s drug-adverse event plots. (A) A bar plot of the number of male-biased drug-adverse event pairs with a drug with ADRA1C as drug target for each System Organ Class (SOC) adverse event term. (B) A bar plot of the number of male-biased drug-adverse event pairs with a drug with ADRA1C as drug target for each Preferred Term (PT) adverse event term. (C) A bar plot of the number of female-biased drug-adverse event pairs with a drug with ADRA1C as drug target for each System Organ Class (SOC) adverse event term [file 40360_2023_727_MOESM16_ESM.png]

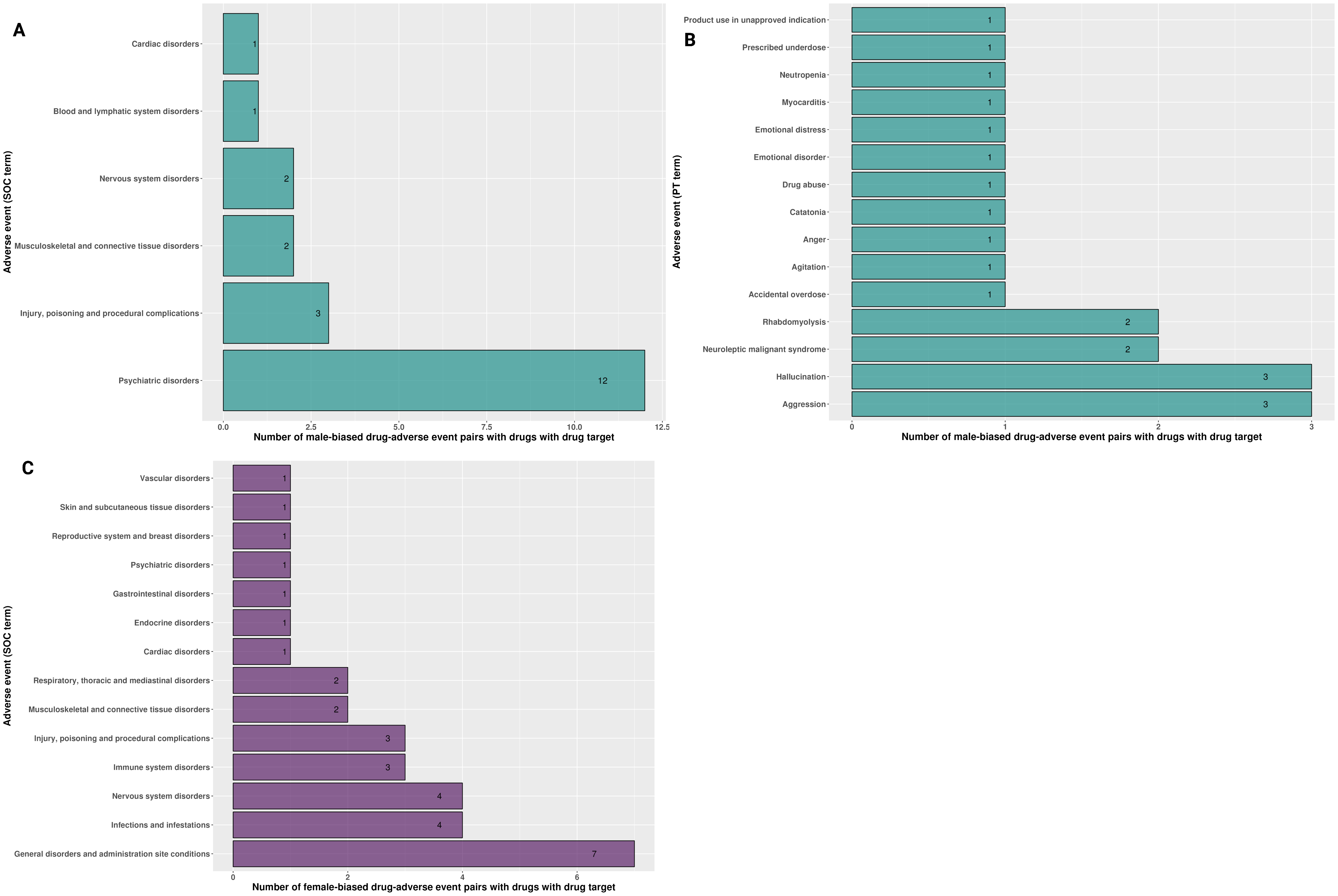

Supplement: Supplementary file 17 — Supplemental Figure 11: Drug target DRD1’s drug-adverse event plots. (A) A bar plot of the number of male-biased drug-adverse event pairs with a drug with DRD1 as drug target for each System Organ Class (SOC) adverse event term. (B) A bar plot of the number of male-biased drug-adverse event pairs with a drug with DRD1 as drug target for each Perferred Term (PT) adverse event term. (C) A bar plot of the number of female-biased drug-adverse event pairs with a drug with DRD1 as drug target for each System Organ Class (SOC) adverse event term [file 40360_2023_727_MOESM17_ESM.png]

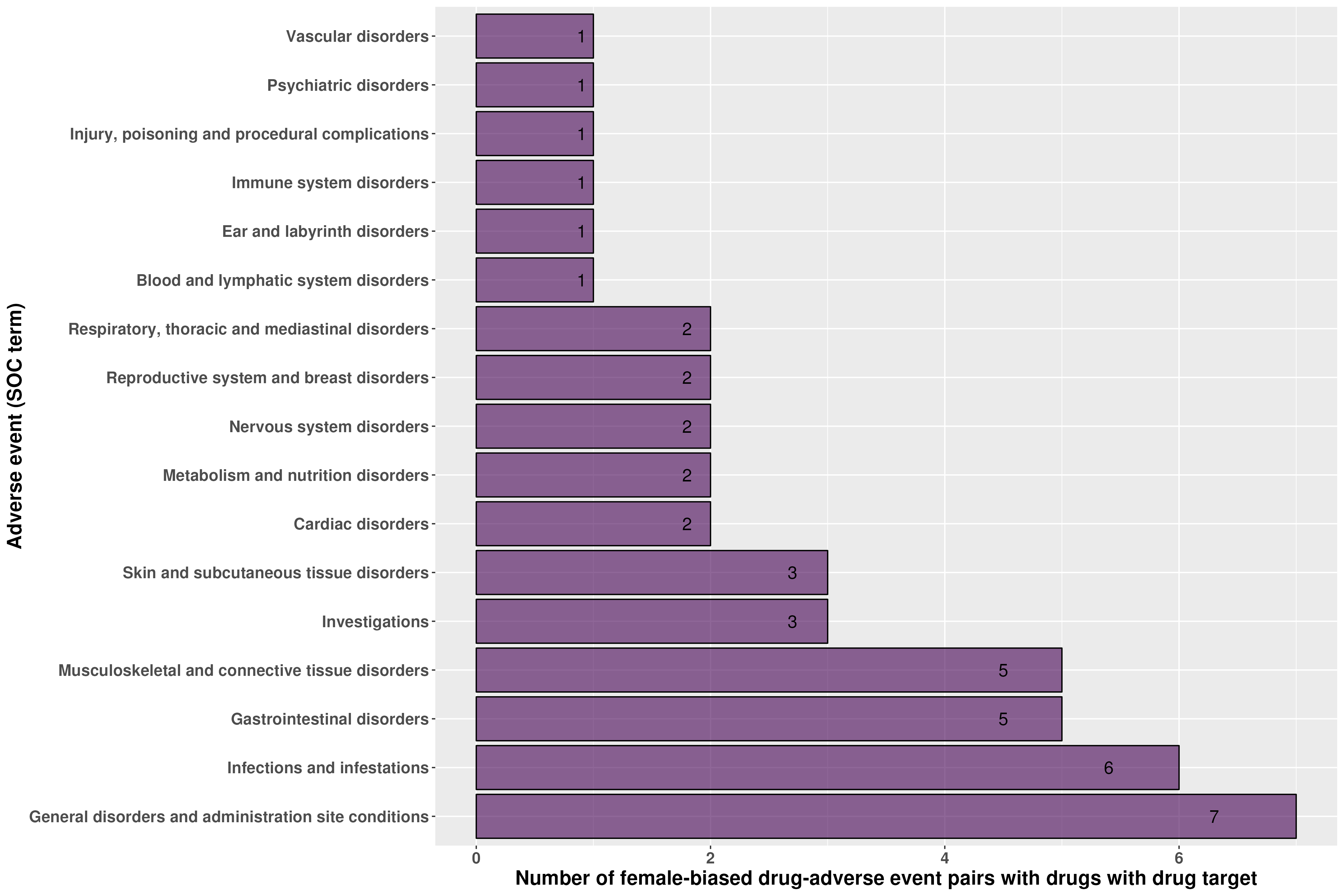

Supplement: Supplementary file 18 — Supplemental Figure 12: A bar plot of the number of female-biased drug-adverse event pairs with a drug with FCGR3B as drug target for each System Organ Class (SOC) adverse event term [file 40360_2023_727_MOESM18_ESM.png]

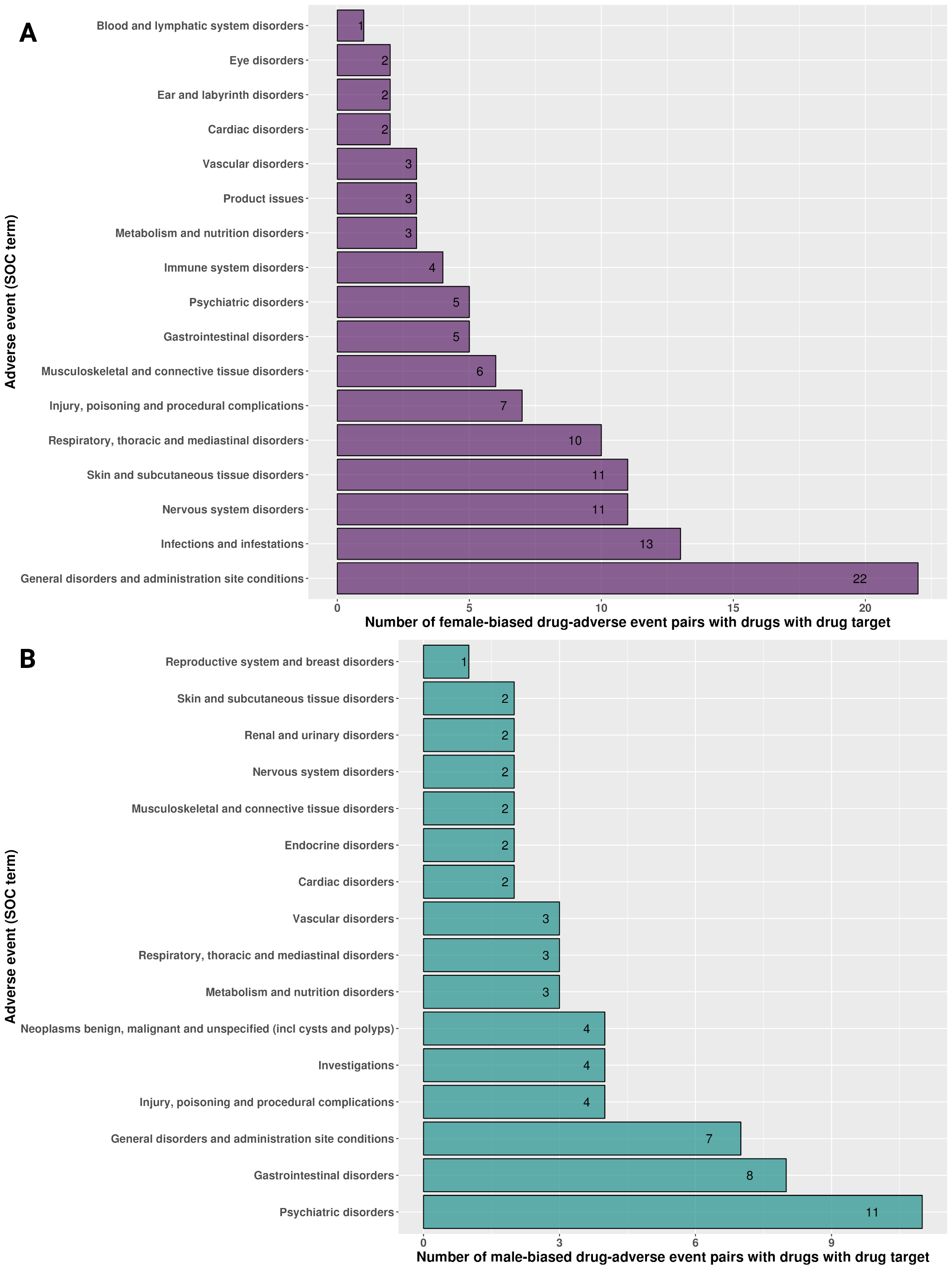

Supplement: Supplementary file 19 — Supplemental Figure 13: Drug target AR’s drug-adverse event plots. (A) A bar plot of the number of female-biased drug-adverse event pairs with a drug with AR as drug target for each System Organ Class (SOC) adverse event term. (B) A bar plot of the number of male-biased drug-adverse event pairs with a drug with AR as drug target for each System Organ Class (SOC) adverse event term [file 40360_2023_727_MOESM19_ESM.png]

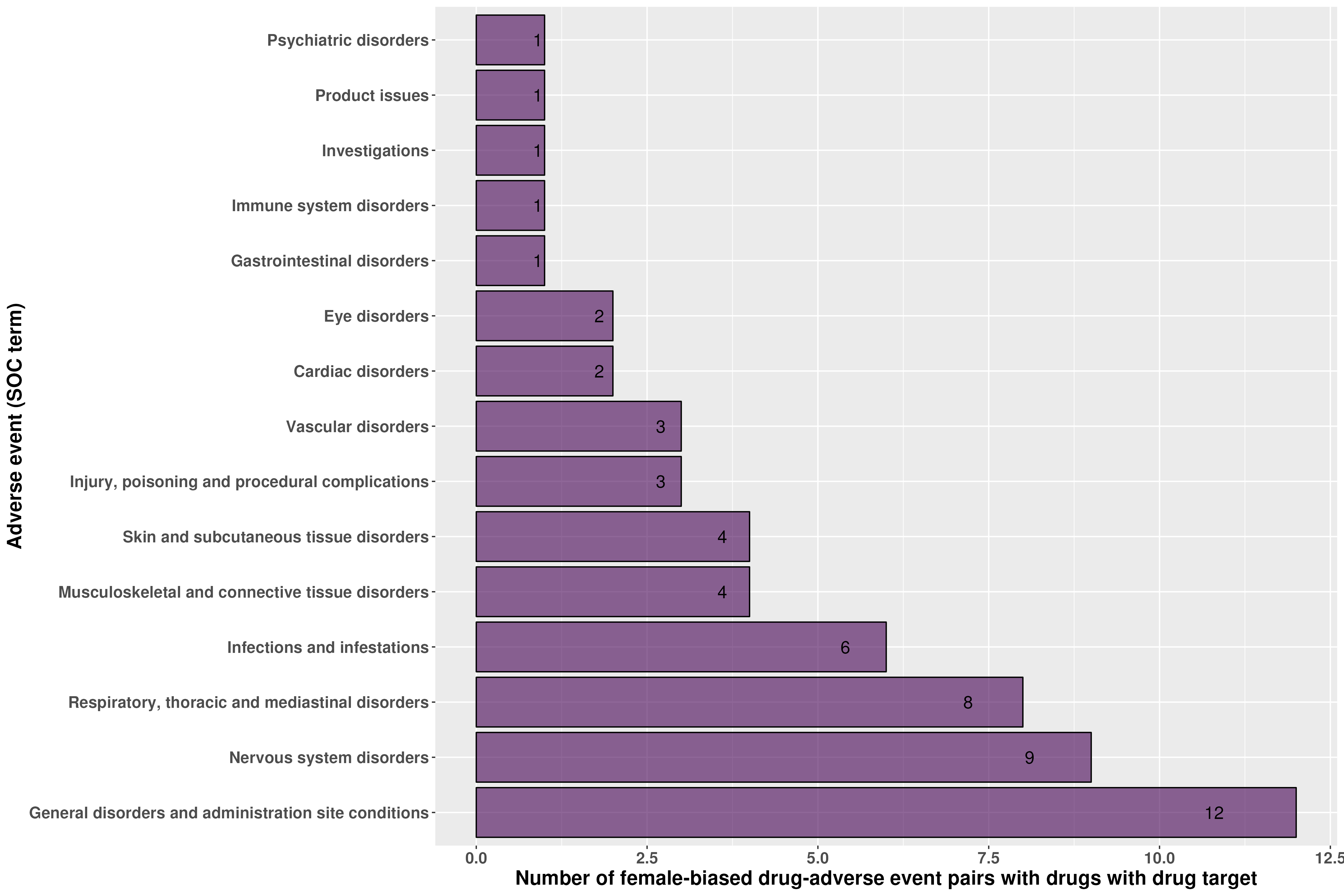

Supplement: Supplementary file 20 — Supplemental Figure 14: A bar plot of the number of female-biased drug-adverse event pairs with a drug with CACNA1S as drug target for each System Organ Class (SOC) adverse event term [file 40360_2023_727_MOESM20_ESM.png]

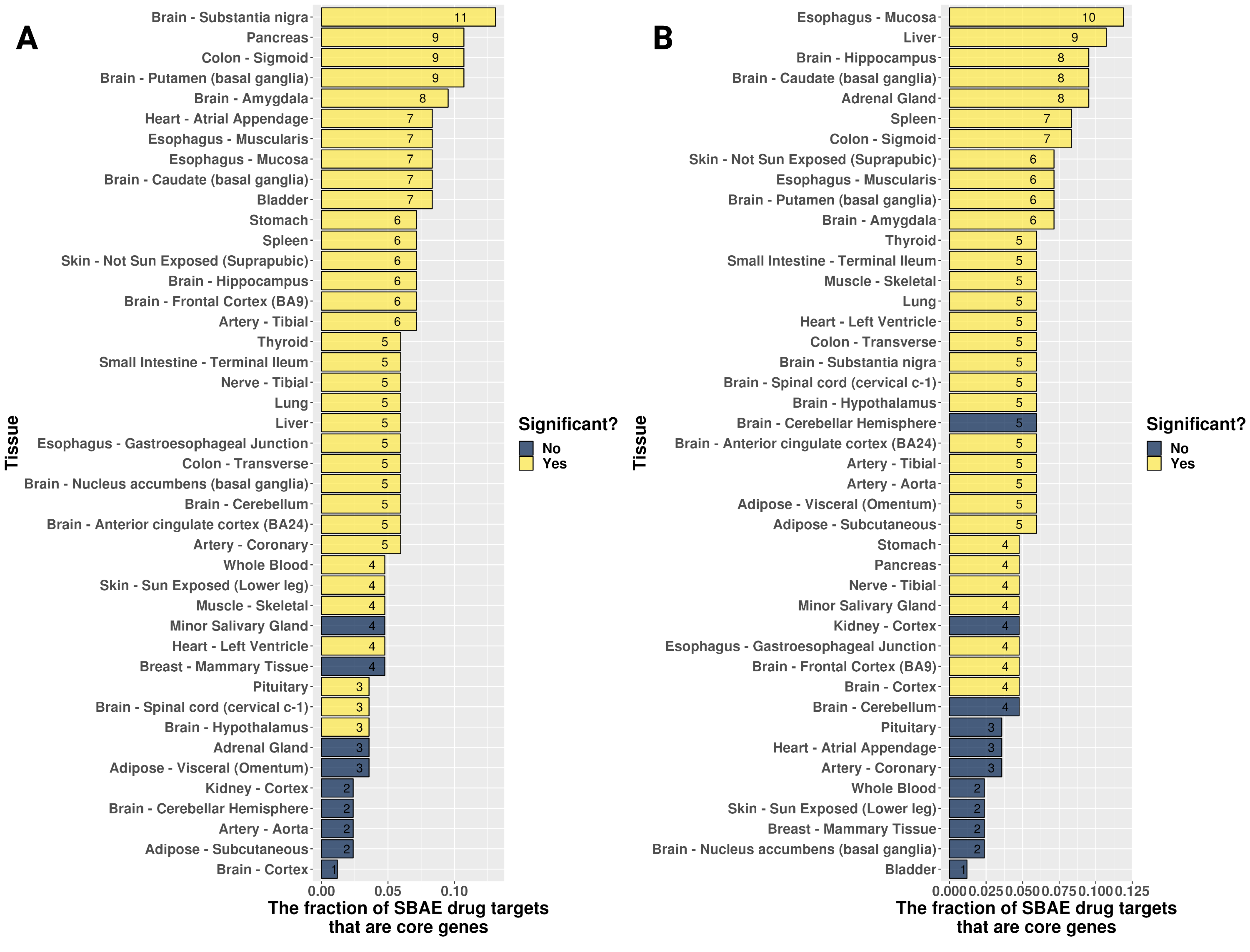

Supplement: Supplementary file 21 — Supplemental Figure 15: Permutation results for the SBAE-associated drug targets’ enrichment of sex-specific network core genes. (A) Bar plot of the female-specific network core gene gene sets by tissue with the x-axis being the fraction of SBAE-associated drug targets with female-specific network core genes for the tissue and the number of drug targets that are female-specific network core genes. (B) Bar plot of the male-specific network core gene gene sets by tissue with the x-axis being the fraction of SBAE-associated drug targets with male-specific network core genes for the tissue and the number of drug targets that are male-specific network core genes. Permutation testing was conducted by randomly selecting either 84 drug targets genes 1,000 times with a one-tailed Wilcoxon test and with BH-multiple hypothesis test correction (α = 0.05) [file 40360_2023_727_MOESM21_ESM.png]
